# Effects of oxytocin on the hair growth ability of dermal papilla cells

**DOI:** 10.1038/s41598-023-40521-x

**Published:** 2023-10-20

**Authors:** Tatsuto Kageyama, Jieun Seo, Lei Yan, Junji Fukuda

**Affiliations:** 1 Faculty of Engineering, Yokohama National University, 79-5 Tokiwadai, Hodogaya-Ku, Yokohama, Kanagawa 240-8501 Japan; 2 Institute of Advanced Sciences, Yokohama National University, 79-5 Tokiwadai, Hodogaya-Ku, Yokohama, Kanagawa 240-8501 Japan; 3 Kanagawa Institute of Industrial Science and Technology, 3-2-1 Sakado Takatsu-Ku, Kawasaki, Kanagawa 213-0012 Japan

**Keywords:** Biomaterials, Regenerative medicine, Tissue engineering

## Abstract

Oxytocin (OXT) is a neuropeptide hormone termed “love hormone” produced and released during childbirth and lactation. It is also produced in response to skin stimulation (e.g., during hugging and massaging) and music therapy. The effects of OXT on various organs have been revealed in recent years; however, the relationship between hair follicles and OXT remains unclear. In this study, we examined the effects of OXT on dermal papilla (DP) cells that control hair growth by secreting growth/regression signals. Gene expression analysis revealed that DP signature markers were significantly upregulated in DP cells treated with OXT. In addition, we tested the hair growth-promoting effects of OXT using in vitro hair follicle organoids. OXT promoted the growth of hair peg-like sprouting by upregulating the expression of growth-promoting factors, including genes encoding vascular endothelial growth factor A (*VEGFA*). This study highlights the positive effects of OXT in hair follicles and may assist in the development of new treatments for alopecia.

## Introduction

People of all ages and sexes are at risk of hair loss caused by genetics, stress, health conditions, and medicines^1, 2^. Current treatments for hair loss include drug therapy^3^, hair transplantation^4^, and stem cell transplantation^5^. Drug therapy is commonly used but is limited by poor treatment outcomes and side effects^6^. Therefore, developing more effective drugs with fewer adverse side effects is crucial.

The hair follicle comprises several parts, the DP, dermal sheath, hair matrix, and outer root sheath^7^. The DP cells at the base of the hair follicle act as the signaling center of hair follicles controlling hair growth by secreting growth/regression signals from the adjacent hair matrix cells^8^. Several growth factors (e.g., vascular endothelial growth factor A (*VEGFA*), platelet-derived growth factor B (*PDGFB*), fibroblast growth factor (*FGF*), and insulin-like growth factor (*IGF*)) secreted by DP cells activate the proliferation and keratinization of hair matrix cells and extend the growth phase of hair follicles^9, 10^. Because the weakness of DP cell functions causes hair loss through reduced hair growth signals, understanding DP activation factors is crucial for developing novel approaches to prevent and treat hair loss.

Hair growth is strongly affected by hormones^11^. Dihydrotestosterone (DHT) is the most potent androgen that modulates hair growth. A double-blinded study showed that DHT levels are significantly higher in bald scalps than hair-containing scalps^12^. Finasteride, an oral drug that can decrease DHT levels, can delay the progression of alopecia and can be used as an effective drug for hair loss treatment^13, 14^. Estrogen and progesterone, sex hormones responsible for various female characteristics in the body, also regulate hair growth^15^. The levels of these hormones increase during pregnancy and cause additional hair growth in the head and body^16, 17^. A recent study has shown that cortisol, a stress-induced hormone, can cause hair loss^18, 19^. The stress hormone produced in the cortex of the adrenal gland inhibits hair regrowth by inhibiting *Gas6* expression in DP^18^.

**Figure 1:**
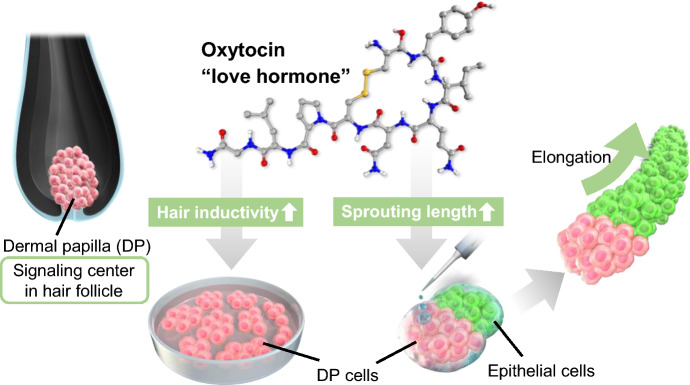
Scheme outlining the identification of oxytocin (OXT) effects on dermal papilla (DP) cells. DP cells and hair follicloids were supplemented with oxytocin for activation and hair growth promotion.

Oxytocin (OXT) is a pregnancy-related hormone secreted during childbirth and lactation. It is sometimes referred to as the “love hormone” because levels of OXT increase during hugging, massaging, music therapy, and interaction with pet dogs^20–22^. Recent studies have suggested that OXT is an anti-stress hormone, and clinical trials have shown that oxytocin provides therapeutic benefits for patients with stress-related disorders^23^. The positive effects of OXT on several organs, including the digestive, muscular, and reproductive systems, have been reported in recent years^24, 25^, but the understanding of the relationship between hair follicles and OXT remains unclear. Herein, we examined the effects of OXT on the hair growth ability of DP cells (Fig. 1). Cultured DP cells were treated with OXT and examined for cell proliferation and DP signature marker gene expression. Finally, the hair growth-promoting ability of OXT was investigated using an in vitro hair growth model. The findings of this study may help in the development of new treatment strategies for hair loss.

## Results

### Effects of OXT on DP cell function

**Figure 2:**
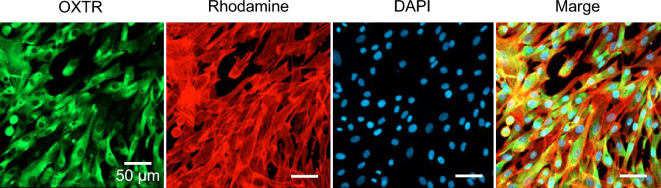
Oxytocin receptor expression in dermal papilla (DP) cells. Cultured DP cells were stained with the anti-oxytocin receptor (OXTR); OXTR (green), nuclei (blue), and actin filaments (red) were visualized using confocal microscopy.

**Figure 3:**
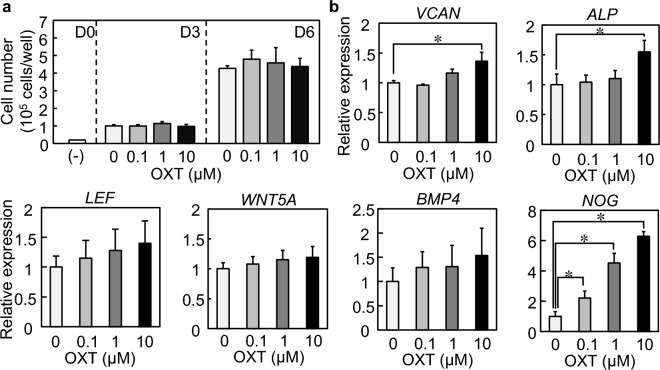
Proliferation and gene expression analysis of dermal papilla (DP) cells cultured in 2D. (**a**) Number of expanded cells at 3 and 6 days of culture. (**b**) Expression of DP signature marker genes. *GAPDH* was used as a reference gene to normalize expression. Error bars represent the standard error of the mean calculated from three experiments for each condition. Using Tukey's test, the numerical variables were statistically evaluated; * indicates *p* < 0.05.

**Figure 4:**
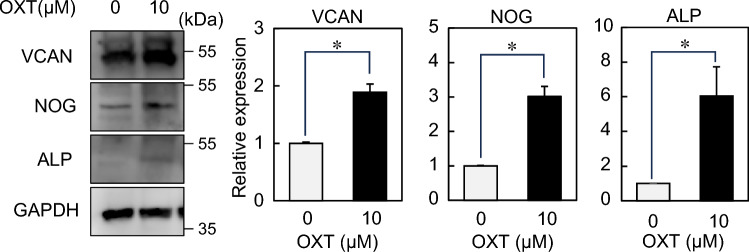
Western blotting analysis of dermal papilla (DP) cells cultured in 2D. Protein expression of VCAN, NOG, and ALP. Proteins in DP cells treated with 0 μM and 10 μM oxytocin (OXT) were analyzed by Western blotting. The intensity of chemiluminescence was normalized with GAPDH. The expression levels are shown as fold changes of the respective values without OXT.

OXT signal transduction begins after OXT binds to the OXT receptor (OXTR). We first confirmed the existence of OXTR for acceptance of OXT signals in DP cells. Immunocytochemical staining and Western blotting revealed that OXTR was expressed in DP cells (Fig. 2, Supplementary Figs. 1, 2). Several other cell sources, including microglia, islet cells, and cardiomyocyte, have OXTR and respond to OXT at 0–10 µM concentration^26–29^. Therefore, OXT treatment at different concentrations (0, 0.1, 1, and 10 µM) was administered to DP cells in 2D culture for 6 days. No significant difference in cell proliferation rate with OXT concentration was observed for 6 days (Fig. 3a). Known markers of DP signature, including versican (*VCAN*), alkaline phosphatase (*ALP*), lymphoid enhancer-binding factor 1 (*LEF1*), Wnt family member 5A (*WNT5A*), bone morphogenetic protein 4 (*BMP4*), and noggin (*NOG*), are generally correlated hair induction and growth capacity^30–33^. These gene expressions were gradually increased with OXT supplementation in a concentration-dependent manner (Fig. 3b). Among them, the levels of *VCAN*, *ALP*, and *NOG* were significantly upregulated by 10 µM OXT treatments (Fig. 3b, Supplementary Fig. 3). The *VCAN*, *ALP*, and *NOG* protein productions were also increased by 10 µM OXT treatments (Fig. 4, Supplementary Fig. 4).

**Figure 5:**
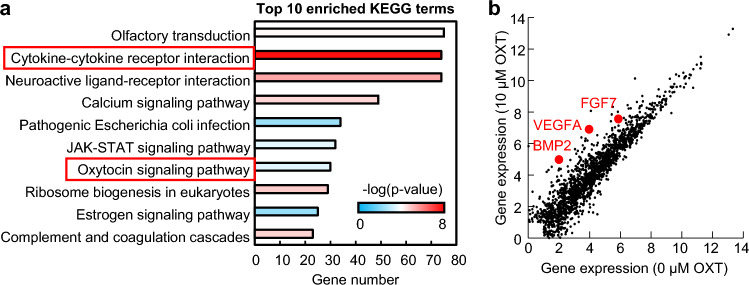
Gene chip analysis and enriched signaling pathway inhibition. (**a**) Kyoto Encyclopedia of Genes and Genomes (KEGG) analysis of upregulated differentially expressed genes (DEGs) showing the top 10 enriched pathways. (**b**) Heat map DEGs between DP cells treated with 0 μM and 10 μM oxytocin (OXT).

We further investigated the key signaling pathways activated by OXT stimulation. DP cells were treated with/without 10 µM OXT for 6 days, and the gene expressions were investigated through comprehensive RNA-seq analysis. RNA-seq results revealed 18,929 DEGs between 0 and 10 µM OXT, of which 8480 genes were upregulated in 10 µM OXT-treated DP. The top 10 enriched pathways represented by these upregulated genes included the cytokine–cytokine receptor interaction and OXT signaling pathway (Fig. 5a). In addition, genes associated with hair growth-promoting factors, including *VEGFA*, *FGF7*, and *BMP2,* were also upregulated by 10 µM OXT treatment (Fig. 5b). These results suggest that mechanisms associated with DP cell activations include cytokine and growth factor secretion through the activation of OXT signaling pathway. Minoxidil, a commercially available hair growth reagent, improves DP cell function to increase the production of growth factors such as *VEGF* and *FGF7*^34, 35^. Since OXT has a similar effect, we hypothesized that OXT might be a candidate for a hair growth reagent and investigated the hair growth ability of OXT using our in vitro hair follicle organoid models^36^.

### Hair growth assay using an in vitro hair growth model

**Figure 6:**
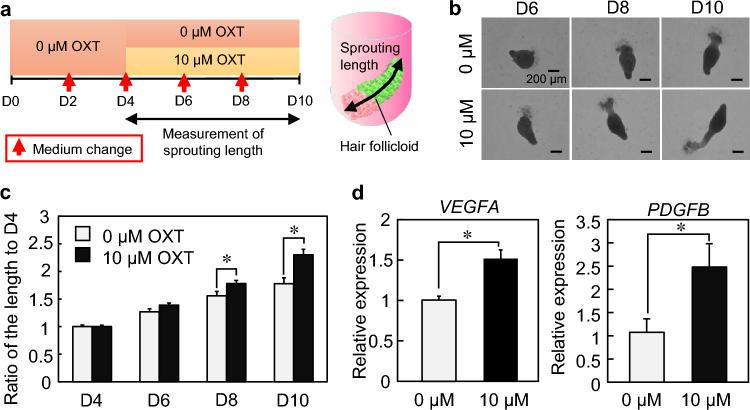
Hair growth testing using hair follicloids. (**a**) Procedures for testing the effects of oxytocin (OXT). (**b**) Microscope images of hair follicloids cultured with/without OXT for 10 days. Hair follicloids were permeabilized and observed using a stereomicroscope. (**c**) The length of sprouting structures with/without OXT. The graph shows the length ratio on days 6, 8, and 10 compared to day 4. (**d**) Relative expression of hair growth-associated genes. *GAPDH* was used as a reference gene to normalize expression. Error bars represent the standard error of the mean calculated from three experiments for each condition. Numerical variables were statistically evaluated using Student’s *t*-test; * indicates *p* < 0.05.

We recently developed an in vitro hair follicle organoid model (termed the hair follicloid) to identify hair growth-promoting factors^36^. In our previous study, hair follicloids were prepared by culturing human DP and epithelial cells in a medium supplemented with a low concentration of Matrigel. Matrigel significantly enhanced the self-organization capabilities of epithelial and mesenchymal cells, resulting in spherical aggregation and subsequent hair peg-like sprouting. Peg-like hair sprouting is composed of hair cortex cell marker AE13 positive cells, but the structure is immature compared with native hair follicles. However, the hair follicloid was sufficient to elongate the peg-like hair sprouting in response to minoxidil. In the present study, we used hair follicloids to examine the effect of OXT on hair growth. Human DP and epithelial cells were suspended in a medium supplemented with 2 v/v% Matrigel and cultured in 96 well spheroid formation plates to prepare hair follicloids. Hair follicloids were treated with 0 and 10 µM OXT after day 4, and hair peg-like sprouting was measured from day 4 to 10 (Fig. 6a). Hair peg-like sprouting was elongated both in 0 and 10 µM OXT-treated hair follicloids. The sprouting length was significantly longer in the 10 µM OXT-treated group than in the untreated control group on days 8 and 10 (Fig. 6b,c). Next, we examined the expression of DP-produced growth factors, including *VEGFA* and *PDGFB*. *VEGFA* and *PDGFB* were significantly upregulated in the presence of OXT (Fig. 6d), suggesting that the elongation of hair peg-like sprouts was promoted by the production of growth factors. These results indicate that OXT has the potential to be a hair growth reagent, although further studies are needed to determine their effects under in vivo environments of hair follicles.

## Discussion

A recent study has shown that OXT accumulates in hair shafts and can be evaluated as a biomarker of stress^37^; however, to the best of our knowledge, there are no studies on the effects of OXT on cells in hair follicles. We found that OXT stimulates DP cells to promote the secretion of growth factors. This finding inspired us to apply OXT therapy to the hair loss treatment, and the hair growth-promoting effects of OXT were confirmed using our in vitro drug screening model. This study revealed the roles of OXT in hair follicles in in vitro, but it would be crucial to understand whether OXT affects hair follicles in more complex in vivo environments for appreciation as a hair loss treatment drug. OXT has been shown to act on multiple cell types in addition to hair follicles^24, 25^, and its use as a treatment for alopecia requires consideration of its side effects on multiple organs. The future study will investigate the understanding of hair growth and side effects using an alopecia model mouse. Also, the present study used DP cells derived from healthy donors, and further investigations must be conducted using cells from patients with alopecia. In addition, understanding age- and sex-dependent efficacy is necessary because OXT production differs with age and gender. OXT concentration and treatment time should be more precisely optimized through these experiments. By advancing these studies, we would like to verify whether OXT can be a new drug for the treatment of alopecia.

The transcriptome analysis of the DP cells revealed that OXT treatment promoted OXT signal transduction and cytokine/growth factor secretion. The DP-secreted factors promoted the growth of hair peg-like sprouting in hair follicloids. However, there are many possible mechanisms by which OXT promotes hair growth. It should be noted that epithelial cells in hair follicloids also have OXTR (Supplementary Fig. 5). Further investigation is needed to understand whether OXT acts directly on epithelial cells to stimulate cell proliferation and growth of hair peg-like sprouting.

Although we investigated the effect of OXT as a hair growth-promoting drug, it may also be used to restore DP cell function in hair regeneration medicine. When DP cells are transplanted with epithelial cells into the skin, DP cells can induce de novo hair follicle regeneration in the recipient's skin^38^. However, the hair regeneration ability of DP cells is gradually lost during expansion culture^39^. We previously developed approaches to restore the hair regeneration ability of DP cells using electrical stimulation and gel bead culturing^40–42^. Combining these methods with OXT may further improve the hair regeneration efficiency of expanded DP cells, which will be examined in future studies.

In conclusion, we showed that OXT activated the DP cells to promote growth factor secretion for hair growth. These findings encourage further investigation for clinical applications of OXT therapy in patients suffering from hair loss.

## Methods

### Preparation of human DP and epithelial cells

Adult human DP cells were obtained from PromoCell (Heidelberg, Germany), passaged up to passage four with R-STEM in hMSC high-growth medium (EM1; Roto, Japan), and used for 2D culture. Adult human follicular keratinocytes (epithelial cells) were obtained from ScienCell Research Laboratories (Carlsbad, CA, USA). DP cells at passage four and epithelial cells at passage one were used for organoid culture. Incubator gas tension was maintained at 21% O_2_ and 5% CO_2_ at 37 °C.

### OXT treatment of DP cells

DP cells (2 × 10^4^ cells) were suspended in 0.5 mL EM1 medium supplemented with 0, 0.1, 1, or 10 μM OXT (Peptide Institute Inc., Japan) and seeded into the wells of a 24-well cell culture plate (Corning Inc., Corning, NY, USA). The culture medium was replaced with a fresh medium every 3 days. The cells were counted using a cell counter (Chemometec, Denmark) after 3 min of trypsin–EDTA treatment. Gene expression in DP cells was assessed using real-time reverse transcription-polymerase chain reaction (RT-PCR) after 6 days of culture.

### OXT treatment of hair follicloids

To investigate the effects of OXT on hair growth, DP cells (5 × 10^3^ cells) and epithelial cells (5 × 10^3^ cells) were suspended in 0.2 mL advanced Dulbecco's Modified Eagle Medium/Nutrient Mixture F-12 (DMEM/F-12; Thermo Fisher Scientific) containing 2% (v/v) Matrigel (Corning Inc.) and seeded into the wells of a non-cell-adhesive round-bottom 96-well plate (Primesurface^®^ 96U plate; Sumitomo Bakelite Co., Ltd., Japan). The DMEM/F-12 medium was supplemented with 10 μM OXT from days 4–10 after seeding. Then, 0.1 mL of the spent medium was replaced with the same fresh medium every 2 days. Hair sprout lengths were observed using an all-in-one fluorescence microscope (BZ-X810, Keyence).

### Immunocytochemical staining

DP cells were cultured with EM1 for 3 days for immunocytochemical staining and fixed with 4% (v/v) paraformaldehyde (FUJIFILM Wako Pure Chemical Corporation, Osaka, Japan) for 10 min. The samples were washed three times with phosphate-buffered saline (PBS) and blocked in blocking solution [PBS containing 3% (v/v) normal goat serum (Abcam Cambridge, UK) and 0.3% (v/v) Triton-X (Sigma Aldrich)] for 1 h at 25 °C. Next, the cells were incubated for 1 h with anti-OXTR (1:200 dilution, 23045-1-AP, Proteintech, Rosemont, IL, USA) at 25 °C. The samples were washed three times with blocking solution and incubated with the corresponding Goat Anti-Rabbit IgG H&L (Alexa Fluor^®^ 488) antibody (1:500 dilution, ab150077, Abcam) in the blocking solution for 1 h at 25 °C and lastly with rhodamine-phalloidin (ab235138, Abcam) and 4′,6-diamidino-2-phenylindole (DAPI; ab228549, Abcam) in PBS for 30 min. A confocal microscope (LSM 700; Carl Zeiss, Germany) was used for fluorescence imaging.

### Gene expression analysis

Total RNA was extracted from the samples using RNeasy Mini Kit (Qiagen, Hilden, Germany) and used for complementary DNA synthesis using the ReverTra Ace^®^ RT-qPCR Kit (Toyobo, Osaka, Japan) according to the manufacturer’s instructions. Subsequent qRT-PCRs were performed using the StepOne Plus RT-PCR system (Applied Biosystems, Waltham, MA, USA) with SYBR^®^ Premix Ex Taq™ II (Takara Bio, Kusatsu, Japan) and primers for amplifying human *ALP*, *VCAN*, *LEF1*, *WNT5A*, *BMP4*, *NOG*, *VEGFA*, *PDGFB*, and glyceraldehyde-3-phosphate dehydrogenase (*GAPDH*) (Table 1). All primers used in this study are listed in Table S1. All gene expression levels were normalized to that of *GAPDH*. The 2^−∆∆Ct^ method was used to determine relative gene expression levels, and they were presented as the mean ± standard error of three independent experiments. Statistical evaluation of numerical variables was conducted using Tukey’s or Student’s t-test, where a p-value of < 0.05 indicated statistical significance.

**Table 1:** PCR primer sequences.

Genes	Forward (5′–3′)	Reverse (5′–3′)
*ALP*	ATTGACCACGGGCACCAT	CTCCACCGCCTCATGCA
*VCAN*	GGCACAAATTCCAAGGGCAG	TCATGGCCCACACGATTAACA
*LEF1*	CTTCCTTGGTGAACGAGTCTG	TCTGGATGCTTTCCGTCAT
*WNT5A*	TCCACCTTCCTCTTCACACTGA	CGTGGCCAGCATCACATC
*BMP4*	GCCCGCAGCCTAGCAA	CGGTAAAGATCCCGCATGTAG
*NOG*	CTGGTGGACCTCATCGAACA	CGTCTCGTTCAGATCCTTTTCCT
*VEGFA*	ACTTCTGGGCTGTTCTCG	TCCTCTTCCTTCTCTTCTTC
*PDGFB*	GAAGGAGCCTGGGTTCCC	TTTCTCACCTGGACAGGT
*GAPDH*	TGGAAGGACTCATGACCACAG	GGATGATGTTCTGGAGAGCCC

### Western blotting

Cell lysates are prepared in RIPA lysis buffer (EzRIOA Lysis kit; ATTO, Tokyo, Japan) as described previously^43^. Briefly, cells were washed with ice-cold PBS and lysed with RIPA lysis buffer. After incubation at 4 °C for 30 min, the cell lysate was centrifuged at 12,000 rpm for 10 min. The supernatant was collected and sequentially mixed with 2 × sodium dodecyl sulfate (SDS) sample buffer. The proteins were separated by electrophoresis on SDS-PAGE gels (Bio-Rad, Hercules, CA, USA) and transferred onto Immobilon-P membranes (Merck KGaA, Darmstadt, Germany). After blocking step with 3% BSA in Tris-buffered saline containing 0.1% Tween-20 (TBST) for 1 h at room temperature, the blots were incubated with primary antibodies (OXTR, 1:1000 dilution, 23045-1-AP, Proteintech; Versican, 1:1000 dilution, PA1-1748A, Thermo Fisher Scientific; ALP, 1:500 dilution, ab229126, Abcam; Noggin, 1:1000 dilution, ab16054, Abcam; GAPDH, 1:2000 dilution, 5174, Cell Signaling Technology) at 4 °C overnight. The membranes were washed with TBST solution three times, then incubated with a horseradish peroxidase-conjugated secondary antibody (1:2000 dilution, Cell Signaling Technology) at room temperature for 1 h. Protein bands on the membrane were visualized using ECL Prime (GE Healthcare, Buckinghamshire, UK) and an Amersham Imager 600 RGB (Cytiva, Tokyo, Japan). The relative protein levels were evaluated using the GAPDH expression level as a reference.

### RNA-seq analysis

Total RNA was extracted using RNeasy Mini Kit (Qiagen) from DP cells with/without 10 μM OXT treatment for 6 days. RNA-seq analysis was performed by Takara Bio. The significantly upregulated genes in DP cells subjected to OXT treatment were used for the Kyoto Encyclopedia of Genes and Genomes pathway analysis with the Database for Annotation, Visualization, and Integrated Discovery (http://david.abcc.ncifcrf.gov/)^44–46^.

### Statistical analysis

Statistical analyses of gene expression levels and the length of hair sprouts were conducted using Tukey’s test or Student’s t-test, and the results were considered statistically significant at *p* < 0.05. All data are presented as mean ± standard error.

### Supplementary Information

Supplementary Information.

## Data Availability

The datasets generated and analyzed during the current study are available in the NCBI repository, GSE233904.
