# Supplementary material for: Effects of oxytocin on the hair growth ability of dermal papilla cells
Source: Sci Rep. 2023 Oct 20;13:15587. doi: 10.1038/s41598-023-40521-x (PMC10589336; doi:10.1038/s41598-023-40521-x)
Supplement: Supplementary file 1 — Supplementary Information. [file 41598_2023_40521_MOESM1_ESM.docx]

**Supplementary information**

**Effects of oxytocin on the hair growth ability of dermal papilla cells**

Tatsuto Kageyama^1,2,3^, Jieun Seo^1,2^, Lei Yan^1,2,3^, and Junji Fukuda^1,2,3^*

^1^ Faculty of Engineering, Yokohama National University, 79-5 Tokiwadai, Hodogaya-ku, Yokohama, Kanagawa 240-8501, Japan

^2^ Institute of Advanced Sciences, Yokohama National University, 79-5 Tokiwadai, Hodogaya-ku, Yokohama, Kanagawa, 240-8501, Japan

^3^ Kanagawa Institute of Industrial Science and Technology, 3-2-1 Sakado Takatsu-ku, Kawasaki, Kanagawa 213-0012, Japan


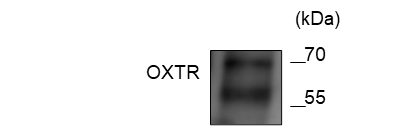


**Supplemental Fig. 1. Expression of oxytocin receptor (OXTR) protein in dermal papilla (DP) cells.** OXTR protein was detected by western blotting.


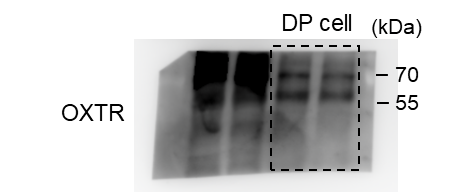


**Supplemental Fig. 2. Original data of western blotting image in Supplemental Fig. 1.**


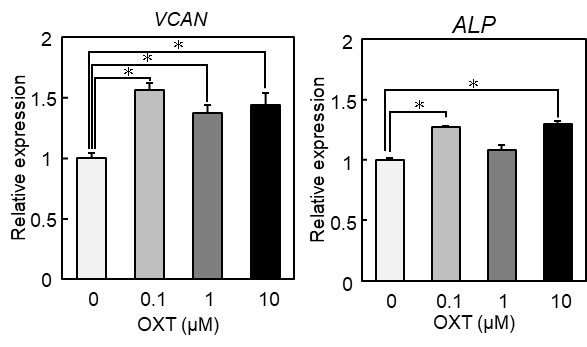


**Supplemental Fig. 3. Expression of DP signature marker genes at 3 days of culture.** *GAPDH* was used as a reference gene to normalize expression. Error bars represent the standard error of the mean calculated from three experiments for each condition. Numerical variables were statistically evaluated using Turkey’s test; * indicates p < 0.05.


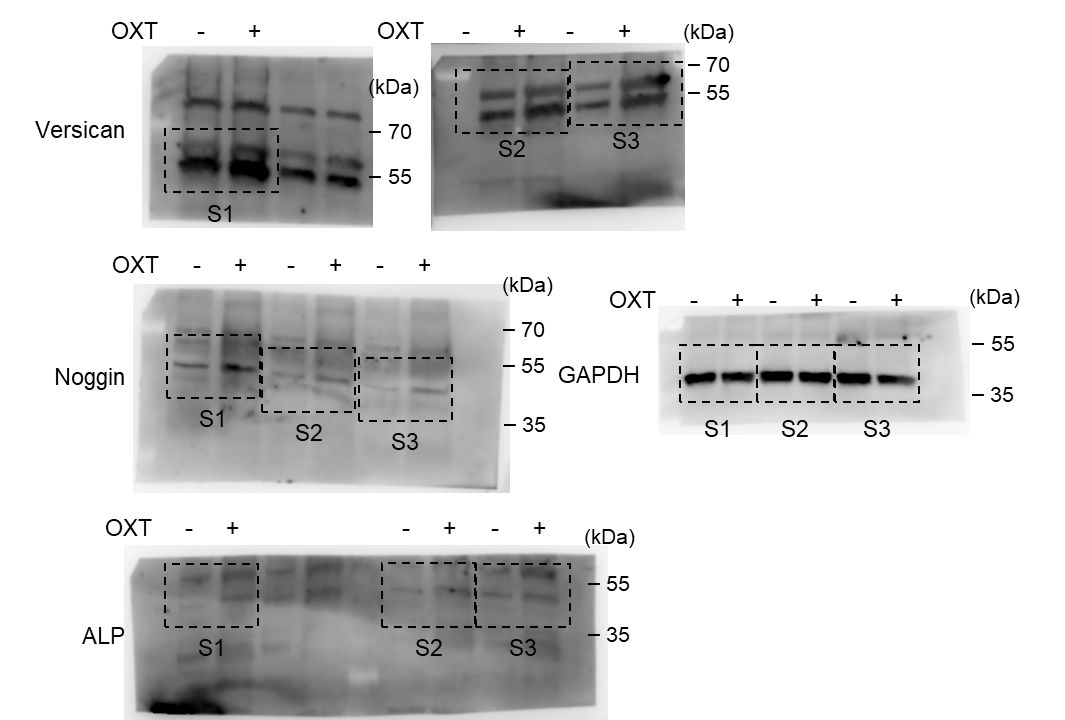
**Supplemental Fig. 4. Original data of western blotting images in Fig. 4.**

**
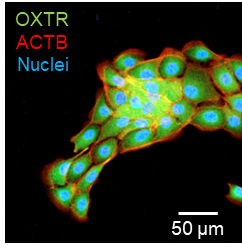
**

**Supplemental Fig. 5. Oxytocin receptor expression in epithelial cells.** Cultured epithelial cells were stained with the anti-OXTR; OXTR (green), nuclei (blue), and actin filaments (red) were visualized using confocal microscopy.
